# Different cardiovascular risk factors are related to distinct white matter hyperintensity MRI phenotypes in older adults

**DOI:** 10.1016/j.nicl.2022.103131

**Published:** 2022-07-29

**Authors:** Jasmin A. Keller, Ilse M.J. Kant, Arjen J.C. Slooter, Simone J.T. van Montfort, Mark A. van Buchem, Matthias J.P. van Osch, Jeroen Hendrikse, Jeroen de Bresser

**Affiliations:** a Department of Radiology, Leiden University Medical Center, the Netherlands; b Department of Intensive Care Medicine and UMC Utrecht Brain Center, University Medical Center Utrecht, Utrecht University, Utrecht, the Netherlands; c Department of Radiology and UMC Utrecht Brain Center, University Medical Center Utrecht, Utrecht University, Utrecht, the Netherlands; d Clinical Artificial Intelligence Implementation and Research Lab (CAIRELab) and Department of Information Technology & Digital Innovation, Leiden University Medical Center, the Netherlands; e Department of Neurology, UZ Brussel and Vrije Universiteit Brussel, Brussels, Belgium

**Keywords:** MRI, Magnetic Resonance Imaging, WMH, White Matter Hyperintensity, SVD, Small Vessel Disease, BMI, Body Mass Index, HADS, Hospital Anxiety and Depression Scale, MMSE, Mini-Mental State Examination, ASA, American Society of Anesthesiologists, FLAIR, Fluid-attenuated Inversion Recovery, SD, Standard Deviation, TIA, Transient Ischemic Attack, CVA, Cerebrovascular Accident, Cardiovascular risk factors, Cerebral small vessel disease, Cognitive decline, Magnetic resonance imaging, White matter hyperintensities

## Abstract

The underlying mechanisms of the association between cardiovascular risk factors and a higher white matter hyperintensity (WMH) burden are unknown. We investigated the association between cardiovascular risk factors and advanced WMH markers in 155 non-demented older adults (mean age: 71 ± 5 years). The association between cardiovascular risk factors and quantitative MRI-based WMH shape and volume markers were examined using linear regression analysis. Presence of hypertension was associated with a more irregular shape of periventricular/confluent WMH (convexity (B (95 % CI)): −0.12 (−0.22–−0.03); concavity index: 0.06 (0.02–0.11)), but not with total WMH volume (0.22 (−0.15–0.59)). Presence of diabetes was associated with deep WMH volume (0.89 (0.15–1.63)). Body mass index or hyperlipidemia showed no association with WMH markers. In conclusion, different cardiovascular risk factors seem to be related to a distinct pattern of WMH shape markers in non-demented older adults. These findings may suggest that different underlying cardiovascular pathological mechanisms lead to different WMH MRI phenotypes, which may be valuable for early detection of individuals at risk for stroke and dementia.

## Highlights

• White matter hyperintensity (WMH) shape: a novel, advanced MRI marker.
• Hypertension correlates with a more irregular shape of periventricular/confluent WMH.
• Different cardiovascular/pathological mechanisms lead to distinct WMH MRI phenotypes.

## Graphical abstract

**Figure:**
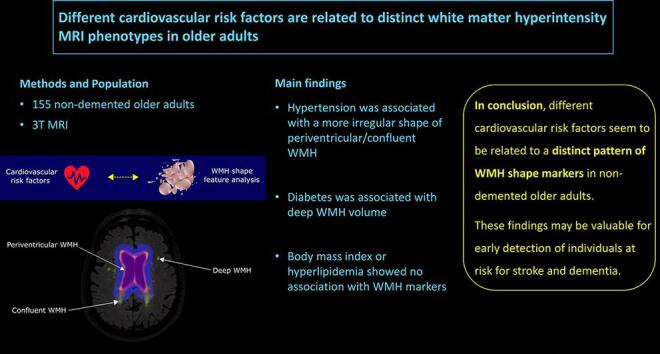

## 1 Introduction

Cerebral small vessel disease (SVD) is associated with the occurrence of dementia and stroke (Jellinger and Attems, 2015). In SVD, different underlying pathological mechanisms (such as small or large vessel atheromas or embolisms) lead to the phenotype of MRI-visible brain changes, namely white matter hyperintensities (WMH), lacunes and microbleeds (Wardlaw et al., 2013a, Wardlaw et al., 2013b).

Individual cardiovascular risk factors play an important role in the etiology of SVD as they impact the small vessels via different pathological pathways and hence lead to distinct patterns of SVD related brain changes that may be differentiated (Alber et al., 2019). An example of this principle is the association between hypertension and deep cerebral microbleeds versus cerebral amyloid angiopathy and lobar microbleeds (Gao et al., 2018). Although WMH are the key MRI marker of idiopathic SVD, little is known about the association of individual cardiovascular risk factors and distinct patterns of WMH.

WMH volume is typically used to study WMH, but this marker fails to fully quantify the complex brain changes related to underlying pathological changes of SVD (Alber et al., 2019). Moreover, WMH volume alone is insufficient for differentiation of underlying disease mechanisms leading to WMH. Aiming to overcome the limitations of conventional WMH volume markers, in recent studies WMH type and shape were introduced as more advanced WMH markers (De Bresser et al., 2018, Ghaznawi et al., 2019). For example, different WMH type (periventricular, deep and confluent) and also different WMH shape is associated with different underlying pathological changes (Alber et al., 2019, Fazekas et al., 1993, Kim et al., 2008). Furthermore, previous studies on WMH shape show potential diagnostic and prognostic value related to an increased mortality and stroke risk (De Bresser et al., 2018, Ghaznawi et al., 2019; Ghaznawi et al., 2021). The investigation of WMH type and shape markers may therefore help in the postulation of potential mechanisms of WMH development. Our hypothesis is that in older adults specific cardiovascular risk factors relate to distinct patterns of WMH, which can be quantified using advanced WMH MRI markers. Studying this hypothesis may aid in the understanding of WMH development and could in the future be used for earlier detection of individuals at risk for stroke or dementia. Therefore, we aimed to investigate the association between cardiovascular risk factors and advanced WMH markers (shape, type, and volume) in non-demented older adults.

## 2 Material and methods

### 2.1 Participants

We included data from the *BioCog consortium* study, collected from the University Medical Center Utrecht site (Winterer et al., 2018). Inclusion criteria for the *BioCog* study were: minimal age of 65 years, a mini-mental state exam (MMSE) score of 24 or higher, and major surgery scheduled of at least 60 min (cardiothoracic (n = 35), gastroenterological (n = 22), gynecologic (n = 6), jaw (n = 11), ear nose throat (13), orthopedic (43), urological (25)). The MMSE was used as a screening for dementia in order to exclude severely cognitively impaired participants. The study was approved by the medical ethics committee (Medical Ethical Committee Utrecht nr. 14/469). Written informed consent was obtained from all participants.

### 2.2 Procedure and demographics

All participants were invited to the hospital before surgery for questionnaires and MRI scanning. Demographic data and medical history questionnaires, and MMSE scores were obtained prior to surgery. Symptoms of anxiety and depression were assessed using the hospital and depression scale (HADS). Scores equal to or above 8 on the depression subscale were considered indicative of depressive symptoms (Zigmond and Snaith, 1983). American Society of Anesthesiologists (ASA) classification scores were performed before surgery by anesthesiologists in training.

### 2.3 Cardiovascular risk factors

Demographic data and data concerning cardiovascular risk factors was collected using questionnaires and medical history records. Diabetes (type I&II), hypertension and hyperlipidemia were registered in the database if they were known by the subject, and treated. BMI in kg/m^3^ was collected as an additional cardiovascular risk factor. Obesity was defined as a BMI equal to or above 30 kg/m^3^.

### 2.4 MRI scans

The scans were performed before surgery on a Philips Achieva 3 Tesla MRI system. The standardized MRI scan protocol included a 3D T1-weighted sequence (voxel size = 1.0 × 1.0 × 1.0 mm^3^; TR/TE = 7.9/4.5 ms) and a 3D fluid-attenuated inversion recovery (FLAIR) sequence (voxel size = 1.11 × 1.11 × 0.56 mm^3^; TR/TE/TI = 4800/125/1650 ms).

### 2.5 WMH volume, type and shape

**Fig. 1:**
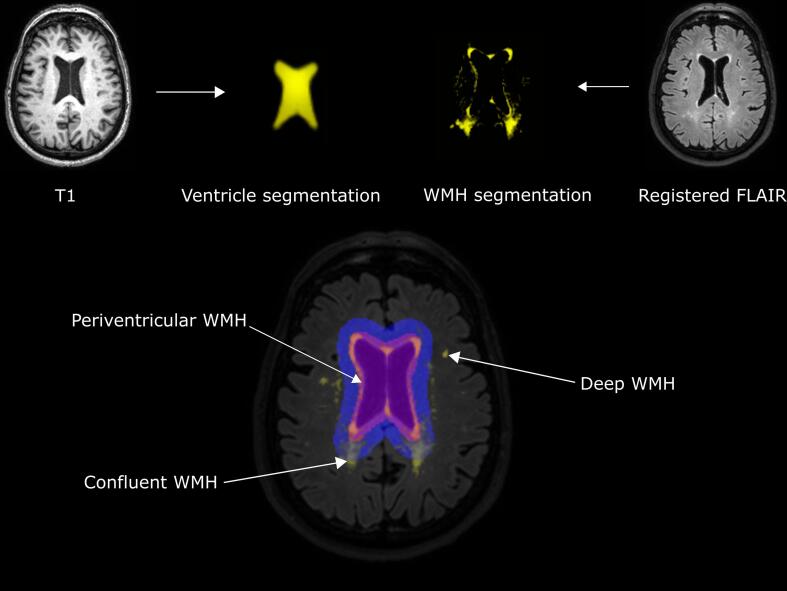
**Schematic illustration of the image processing pipeline.** The lateral ventricles were segmented from the T1-weighted MRI images. WMH segmentation was performed using the registered FLAIR images. WMH were subsequently classified into three types (deep, periventricular, and confluent) using two different masks based on the distance from the ventricles. Periventricular WMH were defined as WMH contiguous with the lateral ventricles and extending ≤ 10 mm into the deep white matter. Confluent WMH were defined as WMH contiguous with the lateral ventricles and extending > 10 mm into the deep white matter. Deep WMH were defined as being located > 3 mm from the lateral ventricles and > 5 voxels (3.45 × 10^−3^ ml). Based on the WMH type, different WMH shape markers were calculated.

Two categories of WMH were automatically determined: ‘deep’ WMH, and ‘periventricular/confluent’ WMH. WMH volumes were calculated automatically using validated methods (Ghaznawi et al., 2019). Based on the WMH segmentation data, the mean values per WMH shape marker (solidity, convexity, concavity index, fractal dimension for periventricular/confluent WMH; fractal dimension and eccentricity for deep WMH) were calculated for each participant with an in-house developed method (Ghaznawi et al., 2019). The image processing pipeline is illustrated in Fig. 1.

Statistical parametric mapping (SPM version 12; Wellcome Institute of Neurology University College London, UK, http://www.fil.ion.ucl.ac.uk/spm/doc/) in Matlab (The MathWorks, Inc., Natick, Massachusetts, United States) was used to register 3D FLAIR images to 3D T1-weighted images. Intracranial volume was calculated using the SPM12 with the option ‘tissue volumes’. The lesion prediction algorithm of the lesion segmentation toolbox version 2.0.15 (Schmidt, 2017; https://www.statistical-modeling.de/lst.html) for SPM12 was used for WMH segmentation. The quality of the WMH segmentations was visually checked by a trained researcher (IK) under supervision of a neuroradiologist experienced in brain segmentation (JB). Cortical brain infarcts were manually delineated and removed from the WMH probability maps. The lateral ventricles were segmented on the T1-weighted images using the automated lateral ventricle delineation toolbox (ALVIN; https://www.nitrc.org/projects/alvin_lv/ (Kempton et al., 2011)) in SPM8. A threshold of 0.10 was applied on the probabilistic WMH segmentations. Periventricular WMH were defined as WMH contiguous with the lateral ventricles and extending ≤ 10 mm into the deep white matter. Confluent WMH were defined as WMH contiguous with the lateral ventricles and extending > 10 mm into the deep white matter. Deep WMH were defined as being located > 3 mm from the lateral ventricles and > 5 voxels (3.45 × 10^−3^ ml). Based on the binary WMH segmentation data, WMH shape markers were calculated (Ghaznawi et al., 2019). For periventricular/confluent WMH, solidity was calculated by dividing lesion volume by the volume of its convex hull. Dividing the convex hull surface area by the lesion surface area provides the convexity of the WMH. A lower solidity and a lower convexity indicates a more complex shape (Ghaznawi et al., 2019). The concavity index is a measure of roughness and was calculated from solidity and convexity (Ghaznawi et al., 2019). A higher concavity index indicates a more irregular shape (Liu et al., 2015). Eccentricity of deep WMH was calculated by dividing the minor axis of the WMH by its major axis. Fractal dimension was calculated as a measure for irregularity of deep, and periventricular/confluent WMH using the box-counting method. Higher fractal dimension and eccentricity values suggest a more complex WMH shape (Ghaznawi et al., 2019). These WMH shape markers were selected because of their ability to capture the expected shape variations of different types of WMH accurately (see Supplementary Table 1 for an overview) (Ghaznawi et al., 2019). Mean values per WMH shape marker were calculated per patient and used for further analyses. The accuracy of the WMH shape marker calculation is dependent on WMH size. Periventricular/confluent lesions vary in size and especially in earlier stages the lesions are not only smaller, but are in a different range of shape markers. The shape parameters used in our study are more accurate in capturing the WMH shape of bigger periventricular/confluent lesions. Therefore a minimum WMH volume of 4 ml was applied as a threshold for the analyses of periventricular/confluent WMH shape markers to assure our method is robust and accurate.

### 2.6 Statistical analysis

Normality of data was assessed by visual inspection of histograms and Q-Q plots. WMH volumes, solidity and concavity index were multiplied by 100 and natural log transformed to correct for non-normal distribution. The statistical analyses performed in this study are exploratory. Associations between cardiovascular risk factors (diabetes, hypertension, BMI, hyperlipidemia) and WMH markers (volume, type, shape) were assessed by linear regression analyses adjusted for age and sex; while WMH volume was additionally adjusted for intracranial volume. In secondary analyses, we performed stepwise linear regression analyses to investigate which of the cardiovascular risk factors accounts for most of the variation of the significantly associated WMH markers. In further secondary analyses, associations between age and sex with the WMH markers were assessed by linear regression analyses; while WMH volumes were adjusted for intracranial volume. Statistical analysis was performed in SPSS version 25. A p value of < 0.05 was considered as statistically significant.

## 3 Results

A total of 178 participants met the inclusion criteria and were included, of which 23 participants had to be excluded from the current study for the following reasons: WMH segmentation errors (n = 2), over-segmentation of WMH (n = 8), anatomic abnormalities (n = 2), missing scans (n = 3), a large cyst (n = 1), MRI motion artefacts (n = 4) or other artefacts (n = 3). Baseline characteristics of the remaining 155 participants are shown in Table 1. A total of 47 % of the participants had hypertension, 34 % had hyperlipidemia, 16 % had diabetes and the mean BMI was 27 ± 4 kg/m^2^.

**Table 1:** Baseline characteristics of the patient population.

	Total (n = 155)
Age	71 ± 5
Female sex	50 (32 %)
MMSE	29 (27,30)
ASA score	
1	19 (12 %)
2	84 (54 %)
3	52 (34 %)
Depressive symptoms	7 (5 %)
Vascular risk factors	
Hypertension	72 (47 %)
Diabetes (type I & II)	24 (16 %)
BMI (kg/m^2^)	27 ± 4
Hyperlipidemia	55 (34 %)
Obesity	30 (19 %)
Current smoker	13 (8 %)
Prior TIA or CVA	8 (5 %)

Data represent n (percentage), mean ± SD or median (interquartile range). MMSE: mini mental state exam; ASA: classification of disease severity for the American Society of Anesthesiologists. BMI: body-mass index. TIA: transient ischemic attack. CVA: cerebrovascular accident.

Presence of hypertension was associated with a more irregular shape of periventricular/confluent WMH (a lower convexity: B (95 % CI): −0.12 (−0.22–−0.03); p = 0.01; a higher concavity index: 0.06 (0.02–0.10); p = 0.01) (see Table 2), but not with total WMH volume (0.22 (−0.15–0.59); p = 0.24).

**Table 2:** The association between cardiovascular risk factors and WMH shape markers.

	Hypertension	Diabetes	BMI	Hyperlipidemia
Periventricular/Confluent WMH^†^				
Solidity^‡^	0.84 (−0.10–0.27)	−0.02 (−0.24–0.20)	0.00 (−0.02–0.02)	−0.01 (−0.18–0.19)
Convexity	−0.12 (−0.22–−0.03)*	−0.03 (−0.15–0.09)	0.00 (−0.01–0.01)	0.01 (−0.09–0.11)
Concavity index^‡^	0.06 (0.02–0.11)*	0.01 (−0.04–0.06)	−0.00 (−0.01–0.01)	−0.01 (−0.05–0.04)
Fractal dimension	0.04 (−0.01–0.10)	0.01 (−0.07–0.07)	0.00 (−0.01–0.01)	−0.03 (−0.08–0.03)
Deep WMH				
Eccentricity	0.01 (−0.03–0.06)	0.00 (−0.05–0.06)	0.00 (−0.01–0.01)	0.02 (−0.02–0.06)
Fractal dimension	0.01 (−0.10–0.12)	−0.03 (−0.17–0.10)	−0.01 (−0.02–0.00)	−0.08 (−0.17–0.03)

The values represent B values (95 % confidence interval) of the linear regression analyses adjusted for age and sex. Periventricular WMH were defined as WMH contiguous with the lateral ventricles and extending ≤ 10 mm into the deep white matter. Confluent WMH were defined as WMH contiguous with the lateral ventricles and extending > 10 mm into the deep white matter. Deep WMH were defined as being located > 3 mm from the lateral ventricles and > 5 voxels (3.45 × 10^−3^ ml). * p < 0.05. ^‡^ Solidity and concavity index were multiplied by 100 and natural log transformed, due to non-normal distribution. ^†^ Periventricular/confluent WMH with a volume > 4 ml. Periventricular/confluent WMH: n = 73; Deep WMH: n = 122.

Presence of diabetes was associated with a higher deep WMH volume (B (95 % CI): 0.97 (0.25–1.70); p = 0.02) (see Table 3). Trends were found for an association between the presence of diabetes and a higher total WMH volume (0.45 (−0.05–0.95); p = 0.07) and a higher perivascular/confluent WMH volume (0.43 (−0.07–0.92); p = 0.09). No associations were found between presence of diabetes and WMH shape markers (see Table 2).

**Table 3:** The association between cardiovascular risk factors and WMH volume.

	Hypertension	Diabetes	BMI	Hyperlipidemia
Total WMH volume	0.22 (−0.15–0.59)	0.45 (−0.05–0.95)	0.00 (−0.04–0.05)	0.14 (−0.24–0.52)
Periventricular/confluent WMH volume	0.21 (−0.16–0.57)	0.43 (−0.07–0.92)	0.00 (−0.04–0.05)	0.15 (−0.23–0.53)
Deep WMH volume	0.24 (−0.32–0.80)	0.89 (0.15–1.63)*	0.05 (−0.02–0.11)	−0.08 (−0.65–0.48)

These values represent B values (95 % confidence interval) of the linear regression analyses adjusted for age, sex and intracranial volume. WMH volumes were multiplied by 100 and natural log transformed, due to non-normal distribution. * p < 0.05. WMH: white matter hyperintensities. BMI: body-mass index.

Neither BMI nor hyperlipidemia were associated with WMH volume or shape markers. The mean WMH shape markers stratified for cardiovascular risk factor are shown in Table 4 and the mean WMH volumes values stratified for cardiovascular risk factor can be found in Table 5.

**Table 4:** Mean WMH shape values per cardiovascular risk factor.

	Total	Hypertension	Diabetes	Obesity	Hyperlipidemia
Periventricular/confluent WMH*					
Solidity	0.19 ± 0.08	0.19 ± 0.07	0.19 ± 0.06	0.19 ± 0.07	0.19 ± 0.08
Convexity	1.15 ± 0.21	1.08 ± 0.18	1.15 ± 0.21	1.19 ± 0.12	1.16 ± 0.22
Concavity index	1.19 ± 0.13	1.23 ± 0.14	1.19 ± 0.16	1.15 ± 0.08	1.18 ± 0.13
Fractal dimension	1.83 ± 0.13	1.87 ± 0.14	1.82 ± 0.13	1.80 ± 0.12	1.82 ± 0.13
Deep WMH					
Eccentricity	0.56 ± 0.12	0.57 ± 0.11	0.56 ± 0.10	0.56 ± 0.15	0.57 ± 0.14
Fractal Dimension	1.83 ± 0.28	1.84 ± 0.32	1.80 ± 0.17	1.76 ± 0.40	1.79 ± 0.34

Data are represented as means ± SD. Obesity was defined as a BMI > 30 kg/m^2^. WMH shape markers are given for the total number of individuals, individuals with diabetes (type I & II), individuals with hypertension, obesity or hyperlipidemia, respectively. * Periventricular/confluent WMH with a volume > 4 ml. Periventricular/confluent WMH: total n = 73; hypertension: n = 36; diabetes: n = 16; obesity: n = 16; hyperlipidemia: n = 31. Deep WMH: total n = 122; hypertension: n = 57; diabetes: n = 22; obesity: n = 25; hyperlipidemia: n = 45.

**Table 5:** Mean WMH volumes per cardiovascular risk factor.

	Total	Hypertension	Diabetes	Obesity	Hyperlipidemia
Total WMH volume	7.84 ± 11.37	10.07 ± 13.29	10.14 ± 13.93	6.77 ± 6.74	7.92 ± 10.62
Periventricular/confluent WMH volume	7.53 ± 11.18	9.67 ± 13.09	9.54 ± 13.56	6.34 ± 6.57	7.61 ± 10.38
Deep WMH volume	0.32 ± 0.59	0.40 ± 0.69	0.61 ± 0.93	0.44 ± 0.74	0.31 ± 0.63

Data are represented as mean volumes (ml) ± SD. WMH volumes are given for the total number of individuals, individuals with diabetes (type I & II), individuals with hypertension, obesity or hyperlipidemia, respectively. Obesity was defined as a BMI > 30 kg/m^2^. Total: n = 155; hypertension: n = 72; diabetes: n = 24; obesity: n = 30; hyperlipidemia: n = 55. Missing values: hypertension: n = 3; BMI: n = 5; hyperlipidemia: n = 3.

In secondary analyses, we performed a stepwise linear regression to investigate which of the significantly associated cardiovascular risk factors accounts for most of the variation of the WMH marker. The results of these analyses were in line with our primary linear regression analyses (see the Supplementary results). In other secondary analyses, age was associated with periventricular/confluent WMH shape (convexity, concavity index and fractal dimension) and WMH volumes (see Supplementary Tables 2 and 3). Furthermore, sex was associated with periventricular/confluent WMH shape (solidity).

## 4 Discussion

The present study aimed to investigate the association of cardiovascular risk factors and novel advanced WMH markers in non-demented older adults. We showed that the presence of hypertension was associated with a more irregular shape of periventricular/confluent WMH, but not with WMH volume. Presence of diabetes was associated with deep WMH volume. BMI or hyperlipidemia showed no association with WMH markers in our study.

We found an association between hypertension and a more irregular WMH shape, but did not find an association with WMH volume. An association between hypertension and WMH volume has previously been shown in several large population-based studies focusing on older adults (Marcus et al., 2011, Muller et al., 2014). Accordingly, hypertension is seen as one of the strongest cardiovascular risk factors associated with WMH. In our study—with a smaller sample size than other population-based studies—we did not find such an association. No previous studies have focused on the association between hypertension and WMH shape. The findings of our study might indicate that WMH shape is a more sensitive marker of hypertension-induced brain changes than WMH volume, since WMH shape was associated with hypertension while WMH volume was not. Based on the results of our study, we postulate that hypertension leads to distinct pathological changes within the small vessels of the brain, which manifest as a distinct WMH MRI phenotype. Hypertension has a direct destructive effect on small arteries, arterioles, venules and capillaries in the brain. Progressive pathological changes to the small vessels of the brain, induced by atheromas and micro-embolisms (Gouw et al., 2011, Wardlaw et al., 2013a), may manifest as distinct WMH phenotypes on MRI due to the anatomical macrostructure of the small vessels located around the ventricles in the white matter. This highlights the strong vascular component in WMH development already suggested in previous research (Alber et al., 2019, Wardlaw et al., 2013b). In our study each WMH shape marker that was analyzed represents a different spectrum of shape variations. For example, presence of hypertension is related to the variation in shape of periventricular/confluent WMH represented by a low convexity and high concavity index, but not to variations in shape represented by solidity or fractal dimension. Important to acknowledge in this regard is that convexity and the concavity index are mathematically related to each other. It is difficult to directly translate our described associations on a group-level to an individual patient in a clinical setting. For future translation to clinical practice, artificial intelligence-based models including a combination of several MRI biomarkers are required for more accurate applications in the field of diagnosis or prognosis.

At present, the association between diabetes and WMH volume is not entirely understood. Previous studies in community-dwelling older individuals have shown associations of type 2 diabetes mellitus and total WMH volume (Murray et al., 2005, Saczynski et al., 2009), while other cross-sectional studies in similar study populations failed to show such an association (Den Heijer et al., 2003, Schmidt et al., 2004). A recent systematic review addressing a possible association between type 2 diabetes and total WMH volume shows an association (Wang et al., 2020). The review did not only focus on cross-sectional or cohort studies, but also included case-control, and Mendelian randomization studies. Furthermore, a previous case-control study focused on patients with type 2 diabetes showed a higher eccentricity of deep WMH and a larger number of periventricular/confluent WMH in diabetic patients compared to controls, but no differences in WMH volume (De Bresser et al., 2018). In our study in non-demented older adults, we did not find an association of diabetes with WMH shape markers, but found an association of diabetes with deep WMH volume. No previous studies have explored WMH shape in non-demented older adults, therefore our results cannot be directly compared to other studies. Based on these findings, WMH shape may be more sensitive than WMH volume for hypertension-induced brain changes, but not for diabetes-induced brain changes.

The association between hyperlipidemia and WMH remains ambiguous. In a previous study, an association between high cholesterol levels and a larger WMH burden was found in the general population above 65 years of age (Breteler et al., 1994). Participants in this previous study are quite comparable to our study regarding age and cardiovascular risk factor profile. A previous case-control study focusing on stroke patients has shown a potential protective role of hyperlipidemia, as hyperlipidemia was associated with lower WMH volumes in two independent cohorts (Jimenez-Conde et al., 2010). However, it is unclear if this protective effect is (partially) mediated through the pharmacological treatment of hyperlipidemia with statins (Jimenez-Conde et al., 2010). This previous study has assessed a specific patient population (stroke patients) with a lower mean age in one of the cohorts compared to our study. No previous study has examined the association between hyperlipidemia and WMH shape. In our study we did not find an association between hyperlipidemia and WMH volume, nor shape markers.

Previous studies conducted using UK-biobank data, with a cross-sectional (Ferguson et al., 2020), as well as an observational cohort study design (Morys et al., 2021), showed an association of BMI with WMH volume. This effect may be mediated via low-grade systemic inflammation (Lampe et al., 2019). However, no previous study has examined the association between BMI and WMH shape markers. In our study, we found no associations between BMI and WMH volume or shape markers. It should be noted that mean BMI in the UK-biobank study was similar to our study, while mean age was lower compared to our study (Ferguson et al., 2020).

Little is known about the exact histopathological mechanisms underlying the formation of WMH, mainly due to the small number of pathology studies that have been conducted (Wardlaw et al., 2015). Interestingly, in a histopathological study, structural markers of vascular dysfunction were found to be associated with WMH (Young et al., 2008). More specifically, vascular integrity was shown to be reduced in areas where WMH were present compared to normal appearing white matter (Young et al., 2008). Periventricular, and deep WMH were associated with different underlying neuropathological findings in a study including Alzheimer’s patients, subjects at risk for cerebrovascular disease and healthy controls (Shim et al., 2015). The study found periventricular WMH volume to be correlated with severity of arteriolosclerosis and breakdown of the ventricular lining (Shim et al., 2015). Deep WMH volume, however, was correlated with cerebral hemorrhages and microinfarcts, as well as demyelination (Shim et al., 2015). Depending on the affected cell type (e.g. vascular or parenchymal), the type of pathophysiology and the anatomical structure of the affected areas, WMH shape may be influenced. These differences in WMH etiology may become evident as different WMH shape patterns and locations (for example punctuate versus elongated deep WMH). Some previous studies have also shown a link between WMH shape and underlying histological findings (Fazekas et al., 1993, Kim et al., 2008). Overall, pathological studies confirm the hypothesis that WMH have a heterogenous etiology (Gouw et al., 2011)—with a strong vascular component. Vascular pathologies may lead to parenchymal changes, subsequently leading to distinct WMH phenotypes on MRI. How different WMH lesion and shape patterns develop related to a certain underlying pathology remains to be investigated in future studies, as in our study it is impossible to link WMH shape patterns to specific underlying mechanisms. Novel advanced MRI markers (such as WMH shape) that are more specific than WMH volume, may help in elucidating the link between histopathology and MRI findings.

The strengths of our study are the relatively large sample size, and the application of novel advanced MRI image processing methods. A limitation of our study could be that participants were all scheduled for major elective surgery, and therefore this sample might not be an equivalent of a general population-based group. This could limit the generalizability of our findings to the general population, as this could have led to an underestimation of the associations. Another limitation of our study could be that the cardiovascular risk factors were based on medical records and self-reported rather than objective measurements (e.g. blood pressure, glucose levels and cholesterol/triglyceride levels). Although these risk factors were scored by qualified physicians (anesthesiologists (in training)), we cannot exclude the possibility that some patients may have undiagnosed hypertension, diabetes or hypercholesterolemia. This may partially account for the lack of significant associations between these cardiovascular risk factors and the WMH markers. An additional limitation could be that although most participants had a relatively high MMSE score (median: 29, (IQR: 27,30)), there may have been participants included in this study with mild cognitive impairment. A technical limitation of our method could be that when total WMH volume increases, usually the deep WMH count decreases, as bigger WMH lesions start to overlap with each other. For instance, deep WMH may become part of confluent WMH and only shape markers for periventricular/confluent WMH can be determined. This can partially explain why we found fewer associations with deep WMH shape markers.

In conclusion, we showed that different cardiovascular risk factors seem to be related to a distinct pattern of WMH shape markers in non-demented older adults. These findings may suggest that different underlying cardiovascular pathological mechanisms lead to different WMH MRI phenotypes, which may be valuable for early detection of individuals at risk for stroke and dementia.

## CRediT authorship contribution statement

**Jasmin A. Keller:** Formal analysis, Visualization, Writing – original draft, Writing – review & editing. **Ilse M.J. Kant:** Investigation, Methodology, Formal analysis, Writing – review & editing. **Arjen J.C. Slooter:** Conceptualization, Writing – review & editing. **Simone J.T. van Montfort:** Investigation, Writing – review & editing. **Mark A. van Buchem:** Writing – review & editing. **Matthias J.P. van Osch:** Writing – review & editing, Supervision. **Jeroen Hendrikse:** Writing – review & editing. **Jeroen de Bresser:** Funding acquisition, Conceptualization, Writing – review & editing, Supervision.

## Declaration of Competing Interest

The authors declare that they have no known competing financial interests or personal relationships that could have appeared to influence the work reported in this paper.
