# Supplementary material for: Different cardiovascular risk factors are related to distinct white matter hyperintensity MRI phenotypes in older adults
Source: Neuroimage Clin. 2022 Jul 29;35:103131. doi: 10.1016/j.nicl.2022.103131 (PMC9421504; doi:10.1016/j.nicl.2022.103131)
Supplement: Supplementary data 1 [file mmc1.docx]

**Supplementary material**

**Results: secondary analyses**

In secondary analyses, we performed a stepwise linear regression to investigate which of the cardiovascular risk factors accounts for most of the variation of the WMH marker. The final model in the stepwise linear regression with convexity as dependent variable included hypertension as only significant predictor (-0.14 (-0.23–-0.05); p<0.01). The final model with concavity index as depended variable included hypertension (0.06 (0.02–0.11); p=0.01) and age (0.01 (0.00–0.01); p=0.01) as significant predictors. The final model with deep WMH volume as depended variable included diabetes (0.76 (0.02–0.12); p=0.04) and age (0.06 (0.00–0.12); p=0.04) as significant predictors.

**Supplementary table 1. Definition of WMH shape markers.**

| **Shape marker** | **Formula** | **WMH type** | **Comment** |
| --- | --- | --- | --- |
| Convexity (C) | $C=\frac{Convex Hull Area}{\mathrm{Area}}$ | Periventricular/Confluent WMH | Convexity and Solidity describe how concave or convex the shape is. A maximally convex shape has a convexity and solidity value of 1. The values decrease with a more concave, complex shape. |
| Solidity (S) | $S=\frac{\mathrm{Volume}}{Convex Hull Volume}$ | Periventricular/Confluent WMH |  |
| Concavity Index (CI) | $CI= \sqrt{\left( 2-C \right)^{2}+\left( 1-S \right)^{2}}$ | Periventricular/Confluent WMH | As a measure of roughness, concavity index describes how dense, irregular or elongated and curved a lesion is. Higher CI values suggest a more complex WMH shape. |
| Fractal Dimension (FD) | $FD=\lim_{r\to1} \frac{log(n_{r})}{log(\frac{1}{r})}$  n = number of boxes  r = box size | Periventricular/Confluent WMH  Deep WMH | Textural roughness is measured using the Minkowski-Bouligand dimension (box counting dimension). Higher FD values suggest a more complex WMH shape. |
| Eccentricity (E) | $E= \frac{Minor Axis}{Major Axis}$  Major axis: largest diameter in 3D space.  Minor axis: smallest diameter orthogonal to the major axis. | Deep WMH | Eccentricity assesses the deviation from a circle. The eccentricity of a circle is 1 and the eccentricity of a line is 0. |

**Supplementary table 2. The association between age, sex, and WMH shape markers.**

|  | **Age** | | **Sex** |
| --- | --- | --- | --- |
| Periventricular/Confluent WMH^†^ | |  |  |
| Solidity^‡^ | 0.02 (-0.04–0.00) | | -0.21 (-0.41–-0.01)* |
| Convexity | -0.01 (-0.02–0.00)* | | 0.00 (-0.11–0.11) |
| Concavity index^‡^ | 0.01 (0.00–0.01)** | | 0.02 (-0.03–0.08) |
| Fractal dimension | 0.01 (0.00–0.02)*** | | 0.01 (-0.06–0.08) |
| Deep WMH |  | |  |
| Eccentricity | 0.00 (-0.00–0.00) | | 0.04 (-0.01–0.08) |
| Fractal dimension | 0.00 (-0.01–0.00) | | 0.00 (-0.11–0.11) |

The values represent B values (95% confidence interval) of the linear regression. Participants with periventricular/confluent WMH: n=73 and with deep WMH: n=122. * p<0.05. ** p<0.01. *** p<0.001. ^‡^ Solidity and concavity index were multiplied by 100 and natural log transformed, due to non-normal distribution. ^†^ Periventricular/confluent WMH with a volume >4 ml. WMH: white matter hyperintensities.

**Supplementary table 3. The association between age, sex, and WMH volume.**

|  | **Age** | **Sex** |
| --- | --- | --- |
| Total WMH volume | 0.11 (0.08–0.15)** | 0.20 (-0.34–0.78) |
| Periventricular/confluent WMH volume | 0.12 (0.08–0.15)** | 0.22 (-0.36–0.80) |
| Deep WMH volume | 0.07 (-0.01–0.12)* | -0.08 (-0.88–0.71) |

These values represent B values (95% confidence interval) of the linear regression analyses adjusted for intracranial volume. WMH volumes were multiplied by 100 and natural log transformed, due to non-normal distribution. n=155. * p<0.05. ** p<0.001. WMH: white matter hyperintensities.
